# Inhibition of PP2A activity by H_2_O_2_ during mitosis disrupts nuclear envelope reassembly and alters nuclear shape

**DOI:** 10.1038/s12276-019-0260-0

**Published:** 2019-06-05

**Authors:** Ju-Hyun Ahn, Min-Guk Cho, Seonghyang Sohn, Jae-Ho Lee

**Affiliations:** 1 Department of Biochemistry and Molecular Biology, Ajou University School of Medicine, Suwon, 443-721 South Korea; 2 Genomic Instability Research Center, Ajou University School of Medicine, Suwon, 443-721 South Korea; 3 Department of Biomedical Sciences, The Graduate School of Ajou University, Suwon, 443-721 South Korea; 4 Department of Microbiology, Ajou University School of Medicine, Suwon, 443-721 South Korea

**Keywords:** Nuclear envelope, Mitosis

## Abstract

Many types of cancer cells exhibit abnormal nuclear shapes induced by various molecular changes. However, whether reactive oxygen species (ROS) induce nuclear deformation has not been fully addressed. Here, we show that hydrogen peroxide (H_2_O_2_) treatment induced concentration-dependent alterations in nuclear shape that were abolished by pretreatment with the antioxidant N-acetyl-L-cysteine or by catalase overexpression. Interestingly, treatment with H_2_O_2_ induced nuclear shape alterations significantly more frequently in mitotic cells than in asynchronous cells, suggesting that H_2_O_2_ mainly affects nuclear envelope disassembly and/or reassembly processes. Because protein phosphatase 2 A (PP2A) activity is reported to be involved in nuclear envelope reassembly during mitosis, we investigated the possible involvement of PP2A. Indeed, H_2_O_2_ reduced the activity of PP2A, an effect that was mimicked by the PP1 and PP2A inhibitor okadaic acid. Moreover, overexpression of PP2A but not PP1 or PP4 partially rescued H_2_O_2_-induced alterations in nuclear shape, indicating that the decrease in PP2A activity induced by H_2_O_2_ is specifically involved in the observed nuclear shape alterations. We further show that treatment of mitotic cells with H_2_O_2_ induced the mislocalization of BAF (barrier-to-autointegration factor), a substrate of PP2A, during telophase. This effect was associated with Lamin A/C mislocalization and was rescued by PP2A overexpression. Collectively, our findings suggest that H_2_O_2_ preferentially affects mitotic cells through PP2A inhibition, which induces the subsequent mislocalization of BAF and Lamin A/C during nuclear envelope reassembly, leading to the formation of an abnormal nuclear shape.

## Cancer: the importance of staying in shape

A class of harmful chemical compounds produces morphological abnormalities in the nucleus that may help promote tumor growth. Reactive oxygen species (ROS) are DNA- and protein-damaging molecules that originate both from environmental contaminants and as a byproduct of cellular metabolism or stress. Jae-Ho Lee and colleagues at Ajou University, Suwon, South Korea have now identified a mechanism by which ROS can disrupt the shape and structure of the nucleus. They show that ROS exposure reduces the ativity of an enzyme called PP2A, which is required for the targeted recruitment of proteins that rebuild the membrane envelope surrounding the nucleus after cell division. Perturbations in this envelope can potentially contribute to damage to the chromosomal DNA within the nucleus, creating conditions that can trigger or accelerate the process of tumorigenesis.

## Introduction

The nuclear envelope is a double membrane that surrounds the chromosomes and contains embedded nuclear pore complexes (NPCs). The outer nuclear membrane and the inner nuclear membrane each have their own complement of inserted proteins. Some proteins in the inner nuclear membrane interact with chromosomes or amin proteins, thus connecting the nuclear envelope to the chromosomes or to the nuclear lamina, respectively. The outer nuclear membrane is continuous with the endoplasmic reticulum (ER) and contains several proteins that connect the nucleus to cytoplasmic filament systems and the centriole, potentially contributing to cell polarity and mobility^1–3^.

In many types of cancer cells, the nuclear shape is often abnormal compared with that of normal cells, making nuclear morphology an indispensable criterion in the current pathological assessment of cancer^4,5^. Corresponding changes in the proteins that form the nuclear membrane are also well known and depend on the cancer cell type^6^. Notably, changes in these proteins are being used as cancer biomarkers^7,8^. Interestingly, in ovarian cancer cells, nuclear abnormalities are associated with chromosomal instability and aneuploidy^9^. However, it is unclear whether the nuclear abnormalities observed in cancer cells are the cause or consequence of cancer formation and progression. Moreover, how the nuclear abnormalities in cancer cells are formed is not well known.

Most reactive oxygen species (ROS) are free radicals derived from an oxygen molecule. ROS can be produced by various intra- and extracellular factors and are in balance with intracellular antioxidants such as glutathione. If this balance is disrupted, free radicals can cause damage to DNA, RNA, proteins, and lipids. ROS can also cause genetic instabilities or mutations and can lead to changes in gene expression. All of these consequences of excess ROS are ultimately capable of producing cancers^10^. In addition, cancer cells, which exhibit accelerated metabolism, require high ROS concentrations to maintain their high proliferation rate^11,12^. Interestingly, it has been reported that H_2_O_2_ alters the nuclear shape in ataxia-telangiectasia cells by increasing the amount of Lamin B1 protein, one of the main components of the nuclear lamina^13^. However, the specific relationship between nuclear shape changes and ROS in cancer cells is still not well understood.

During the interphase of the cell cycle, the nuclear membrane remains intact and relatively stable. However, during mitosis, the nuclear envelope changes dramatically. In early mitosis, the nuclear membrane becomes fragmented, and the nuclear envelope and lamina break down (disassembly of the nuclear envelope). Conversely, during mitotic anaphase and telophase, the nuclear structures assemble around the chromosomes to re-form the nuclear envelope (reassembly of the nuclear envelope)^1,3^. Therefore, it is highly likely that the cue that induces abnormal nuclear shapes acts in a cell cycle-dependent manner. Specifically, cells in mitosis may be more vulnerable to the events that affect nuclear shape because this is when the disassembly and reassembly of nuclear envelopes occurs.

Like other mitotic events, these processes are spatially and temporally controlled by the balance of various kinases and phosphatases^3,14,15^. A recent study reported that protein phosphatase 2 A (PP2A) plays an important role in the reassembly of the nuclear envelope during mitotic exit^16^. It is well known that PP2A is inhibited by hydrogen peroxide (H_2_O_2_)^17–19^ and that its activity is decreased in various cancer cell types^20^, suggesting the possible involvement of PP2A in the relationship between ROS and nuclear shape changes.

BAF (barrier-to-autointegration factor) is a protein that binds to the LEM domain of inner nuclear membrane proteins (e.g., LAP2β, Emerin, and MAN1)^21,22^ and acts as a bridge between the nuclear envelope and chromatin by interacting with chromatin^23^. BAF is phosphorylated by VRK1 (vaccinia-related kinase 1) during early mitosis and dephosphorylated by PP2A during mitotic exit^16,24^. Dephosphorylation enables BAF to localize at the chromosomal ‘core’ region, which defines the central region of a set of daughter chromosomes near the spindle pole during telophase^25^. Emerin, LAP2β and Lamin A are recruited to the core region in a BAF-dependent manner during telophase, forming an important structure in the nuclear envelope reassembly process^22,25,26^. Importantly, it has been reported that knockdown of BAF results in nuclear abnormalities^27–29^.

In this study, we show that H_2_O_2_ induces the formation of abnormal nuclei, especially when exposure occurs during mitosis. Our data further suggest that H_2_O_2_ reduces the activity of PP2A, hindering the subsequent proper localization of BAF as well as Lamin A at the core region during nuclear envelope reassembly. These events result in abnormal reassembly of the nuclear envelope during mitotic exit and appear to underlie the subsequent formation of abnormal nuclear shapes.

## Materials and methods

### Antibodies

The following antibodies were used: mouse monoclonal antibodies against α-tubulin (Santa Cruz, sc-23948), BAF (Abnova, H00008815-M07), GAPDH (Santa Cruz, sc-32233), PP2A (BD science, 610555), mAb414 (Covance, MMS-120P), Lamin A/C (Santa Cruz, sc-7292), GFP (Santa Cruz, sc-9996), phospho γ-H2A.X (Ser139) (EMD Millipore, 05-636) [all at a 1:500 dilution in phosphate-buffered saline (PBS) supplemented with 3% BSA] and Flag (Sigma-Aldrich, F1804) at a 1:5000 dilution in PBS supplemented with 3% BSA; and a rabbit polyclonal antibody against Lamin B1 (Abcam, ab16048) at a 1:500 dilution in PBS supplemented with 3% BSA. Horseradish peroxidase-conjugated anti-mouse (G21040) and anti-rabbit (G21234) antibodies were obtained from Invitrogen (used at a 1:5000 dilution in TBST). The following fluorochrome-conjugated secondary antibodies were used (at a 1:500 dilution in PBS supplemented with 3% BSA): anti-mouse Alexa Fluor-488 (Invitrogen, A11059), anti-rabbit Alexa Fluor-488 (Invitrogen, A11034), TRITC-conjugated phalloidin (Jackson Immunoresearch, P1951), anti-mouse Cy3 (Jackson Immunoresearch, 715-165-151), and Alexa Fluor-594 (Invitrogen, A11037).

### Cell culture

HeLa cells were purchased from the American Type Culture Collection (Manassas, VA, USA) and cultured in Dulbecco’s modified Eagle’s medium/nutrient mixture F-12 Ham (DMEM/F12, Sigma-Aldrich, D8900) supplemented with penicillin/streptomycin (Gibco BRL, 15240-062) and 10% (V/V) fetal bovine serum (Gibco BRL, 16000-044). The hTERT-immortalized retinal pigment epithelial cell line, hTERT RPE-1, was obtained from the ATCC and cultured in DMEM/F12 supplemented with penicillin/streptomycin, 10% FBS and 0.01 mg/ml hygromycin B (Sigma-Aldrich, H0654). The human osteosarcoma cell line U2OS was also obtained from the ATCC and cultured in DMEM/high-glucose medium (Gibco, 31600-034) supplemented with penicillin/streptomycin and 10% FBS. HT1080 cells were obtained from the ATCC and cultured in DMEM (Gibco, 31600-026) supplemented with penicillin/streptomycin and 10% FBS. All cells were cultured at 37 °C in a humidified incubator with 5% CO_2_ in air.

### Synchronization and drug treatment

Mitotic cell synchronization was performed by thymidine/RO3306 block as described in a previous paper^30^. Briefly, cells (5 × 10^5^) were seeded into 100-mm culture plates (BD FALCON, 353003), cultured in DMEM/F12 or DMEM containing 10 % FBS for 1 day and subsequently treated with 2 mM thymidine (Sigma-Aldrich, T9250) to arrest at the G1/S transition. 20 h later, the cells were washed with thymidine-free medium and cultured in complete medium for 7 h (for HeLa cells) or 8 h (for U2OS and RPE-1 cells). Then, the cells were cultured again in 9 μM RO3306 (Enzo, ALX-270_463) containing medium to arrest at the G2/M transition for 2 h (for HeLa cells) or 3 h (for U2OS, RPE-1, and HT1080 cells). Mitotic cells were isolated by mechanical shake-off following 30 min of release from RO3306 treatment. The cells were treated with N-acetyl-L-cysteine (NAC, A9165, Sigma-Aldrich, USA) at a final concentration of 10 mM 30 min before H_2_O_2_ treatment, unless otherwise stated.

### H_2_O_2_ treatment

A 10 mM H_2_O_2_ (Sigma-Aldrich, 216763) solution was prepared in distilled water immediately before use and then added to culture media to a final concentration of 50, 100 or 200 μM. The actual concentration of H_2_O_2_ was determined by measuring the OD at 240 nm using a molar extinction coefficient of 43.6 M^−1^ cm^−1^ ^31^.

### Determination of intracellular ROS levels

HeLa cells (1 × 10^5^) were plated on a 60-mm culture plate (Techno Plastic Products, TP93060) and subsequently treated with 2 mM thymidine to arrest at the G1/S transition. 20 h later, the cells were washed with thymidine-free medium and cultured in complete medium for 8 h. Then, the cells were cultured again in medium containing 100 ng/ml nocodazole (Sigma, M1404) to arrest in mitosis for 3 h. Mitotic cells were isolated by mechanical shake-off. The cells were treated with NAC at a final concentration of 10 mM 30 min before H_2_O_2_ treatment. Then, the cells were exposed to hydrogen peroxide solution for 1 h and were then washed with PBS). Cells were then treated with 20 μM dichlorodihydrofluorescein diacetate (DCF-DA) for 20 min. The amounts of intracellular ROS were determined as the fluorescence intensities of DCF-DA with a BD FACSAria™ III using an Argon laser with a 525 nm (DCF-DA) bandpass filter. The data were analyzed with WIN MDI software (Windows Multiple Document Interface for Flow Cytometry: http://facs.scripps.edu/).

### Measurement of intracellular H2O2 levels in living cells

The intracellular level of H_2_O_2_ was measured using the fluorescent sensor pHyper-Cyto^32^; the experimental procedure was described in a previous paper^30^. Briefly, 2 days before the experiment, HeLa cells were transfected with pHyper-Cyto using electroporation, and fluorescence was monitored by using a microscope (a Nikon Eclipse Ti with a 20 × 14 NA Plan Apochromat objective). Images were captured with an iXonEM+897 electron-multiplying charge-coupled device camera. pHyper has two excitation peaks, with maxima at 405 and 488 nm, and one emission peak. Upon reaction with H_2_O_2_, the excitation peak at 405 nm decreases proportionally to the increase in the peak at 488 nm^32^. Therefore, the change in the intracellular H_2_O_2_ level can be evaluated by calculating the pHyper fluorescence ratio upon excitation by 488 and 405-nm lasers. pHyper fluorescence images were acquired in two different channels: CH1 (excitation wavelength, 488 nm) and CH2 (excitation wavelength, 405 nm) with time-lapse imaging. The fluorescence index is expressed as the ratio of CH1 to CH2. Images were acquired at intervals of 30 s for 25 min. A higher index corresponds to higher levels of intracellular H_2_O_2_. Image analysis was performed using NIS elements Ar microscope imaging software.

### Plasmids and transfection experiments

HA-catalase was a gift from Dr. Gyesoon Yoon (Ajou University, Korea). GFP-BAF and GFP-Emerin were from Dr. Yasushi Hiraoka (Osaka University, Japan). Flag-PP4 was a gift from Dr. Daniel Durocher (University of Toronto). CFP-PP2A was gifted from Dr. Hyeseong Cho (Ajou University, Korea) and subsequently subcloned using a gateway system into the Flag-destination or emGFP-destination vectors to generate Flag-PP2A and emGFP-PP2A, respectively. GFP-PP1 (plasmid #44224) was purchased from Addgene. The GFP-BAF S4A mutant was constructed using a Muta-Direct^TM^ site directed mutagenesis kit (iNtRON Biotechnology, #15071). HeLa cells were transfected using Neon electroporation (Invitrogen).

### Transmission electron microscopy

Cells were prefixed in Karnovsky’s solution (1 % paraformaldehyde, 2% glutaraldehyde, 2 mM calcium chloride, and 0.1 M cacodylate buffer; pH 7.4) for 2 h and washed with cacodylate buffer. Postfixing was carried out in 1% osmium tetroxide and 1.5% potassium ferrocyanide for 1 h. After dehydration with 50–100% alcohol, the cells were embedded in Poly/Bed 812 resin (Pelco, Redding, CA, USA), polymerized and observed under an electron microscope (TEM; Zeiss EM 902 A, Zeiss, Oberkochen, Germany).

### Immunocytochemistry

Mitotic cells were split onto poly-L-lysine (PLL, P6282, Sigma-Aldrich)-coated slides. Cells were grown on coverslips and fixed in 4% formaldehyde or 10% trichloroacetic acid (for the analysis of BAF localization) in PBS for 15 min at RT and permeabilized with 0.2% Triton X-100 (Sigma, 9002-93-1) in PBS for 10 min at RT. Fixed cells were preincubated in blocking solution (3% bovine serum albumin in PBS), followed by incubation with primary antibodies at 4 °C overnight. After incubation with primary antibodies, the cells were washed three times in PBS with shaking and probed with fluorescein (Cy3, Alexa Fluor 488, or Alexa Fluor 549)-conjugated anti-mouse or anti-rabbit secondary antibodies. After washing three times with PBS, DAPI (Invitrogen, D3571) was used for DNA counterstaining. Three washes with PBS were followed by mounting in the mounting solution (Biomeda, M01). The samples were examined under a fluorescence microscope (Axio Imager M1, Carl Zeiss).

### Immunoblotting

Conventional immunoblotting was performed as previously described^33^ using the corresponding antibodies. Briefly, cell lysates (30 μg) were resolved by sodium dodecyl sulfate–polyacrylamide gel electrophoresis and were then transferred to polyvinylidene fluoride membranes. After blocking for 1 h at room temperature (RT) with TBS containing 0.1% (V/V) Tween‐20 and 5% (W/V) nonfat milk, membranes were incubated with the corresponding primary antibodies at 4 °C, followed by washing with TBS containing 0.1% Tween-20 and incubation with a horseradish peroxidase-conjugated anti-rabbit or anti-mouse antibody (Amersham Biosciences, Piscataway, NJ) for 1 h at RT. Detection was carried out using ECL reagents (Amersham Biosciences) and exposure of the membranes to X-ray film.

### Protein phosphatase 2A (PP2A) activity assay

Phosphatase activity was determined using the DuoIC set PP2A phosphatase activity kit (R&D Systems, DYC3309-2) according to the manufacturer’s instructions. Cells were rinsed twice with TBS and solubilized in 1 ml of lysis buffer (50 mM HEPES, 0.1 mM EGTA, 0.1 mM EDTA, 120 mM NaCl, and 0.5% Nonidet P-40; pH 7.5, supplemented with 25 μg/ml leupeptin, 25 μg/ml pepstatin, 2 μg/ml aprotinin, and 1 mM PMSF) per 1 × 10^7^ cells. The cell extract was centrifuged at 2000×*g* for 5 min, and the sample protein concentration was quantified using the Bradford assay. Three hundred micrograms of the cell lysate was added to 96-well plates coated with an immobilized capture antibody specific for the catalytic subunit of PP2A. After removing unbound material, a serine/threonine synthetic phosphopeptide substrate, which is dephosphorylated by active PP2A to generate free phosphate and the unphosphorylated peptide, was added. The free phosphate released during the 30 min incubation was then detected by a dye-binding assay using malachite green and molybdic acid. The activity of PP2A was determined by calculating the rate of phosphate release.

### Time-lapse microscopy analysis

For time-lapse live cell imaging, HeLa cells were transfected with GFP-BAF, and seeded (1 × 10^4^) onto a 4-well glass-bottom dish (Thermo Scientific™ Nunc™ Lab-Tek II Chambered* Coverglass, 154526). The cells were treated with 2 mM thymidine (Sigma-Aldrich, T9250) to arrest at the G1/S transition. 20 h later, the cells were washed with thymidine-free medium and cultured in complete medium for 7 h. Then, the cells were cultured again in medium containing 9 μM RO3306 (Enzo, ALX-270_463) to arrest at the G2/M transition for 2 h. The cells were released from RO3306 treatment and stained with Hoechst 33342 for the visualization of chromosomes. After 30 min, the cells were treated with 50 μM H_2_O_2_ or 100 nM okadaic acid. Fluorescence images were acquired every 3 min using a Nikon eclipse Ti with a 20 × 14 NA Plan Apochromat objective. Images were captured with an iXonEM + 897 electron-multiplying charge-coupled device camera and analyzed using NIS elements Ar microscope imaging software.

### Dephosphorylation of BAF by Phosphatases

Cells were cultured in medium containing 100 ng/ml nocodazole (Sigma, M1404) to arrest in mitosis for 16 h. Mitotic cells were isolated by mechanical shake-off. Then, the cell lysates were reacted with lambda phosphatase (NEB, P0753S) in lambda phosphatase buffer (20 mM Tris-HCl, pH 7.6; 250 mM NaCl, and 0.5% NP-40) supplemented with 2 mM MnCl_2_ at room temperature for 2 h; the reaction was stopped with Laemmli sample buffer.

### Knockdown experiments

High-performance liquid chromatography-purified (>97% pure) small interfering RNA (siRNA) oligonucleotides targeting BAF were purchased from Genolution. The sequences of the sense strands of the siRNA oligonucleotides were as follows: BAF, 5′-GACAGUUACCAGCUUUCCUUU-3′; 5′-AGGAAAGCUGGUAACUGUCUU-3′. Cells were transfected with 10 nM of either the BAF1 or control siRNA oligonucleotides using Neon electroporation apparatus (Invitrogen).

### Statistical analysis

Most data are presented as the means ± standard deviations (SDs). Each experiment was performed in triplicate. Statistical differences were analyzed by Student’s t-test, and asterisks (*) and pound signs (^#^) indicate significant differences: *,^#^*P* < 0.05; **,^##^*P* < 0.01; and ***,^###^*P* < 0.001.

## Results

### Treatment of mitotic cells with H2O2 induces the formation of abnormal nuclei

**Fig. 1:**
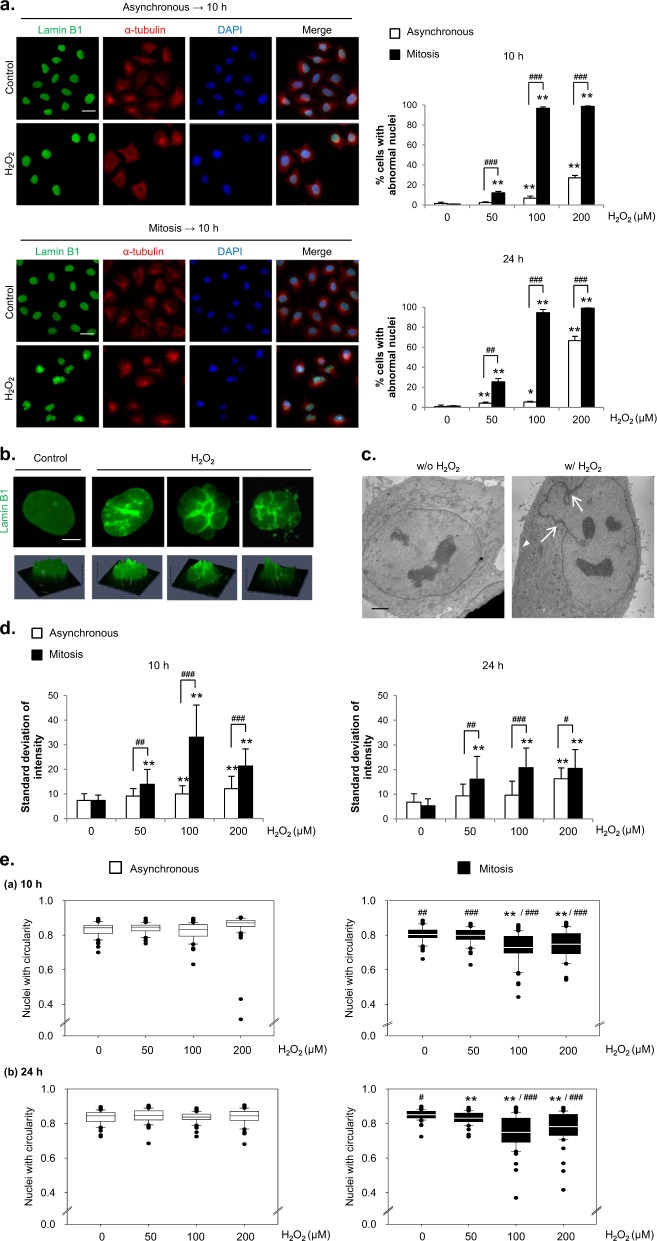
H_2_O_2_ treatment forms a more abnormal nuclear shape in mitotic cells than in asynchronous cells. **a** Left panel: Asynchronous (upper) or mitotic (lower) HeLa cells were treated with 100 μM H_2_O_2_ for 10 h and subjected to immunocytochemical staining for Lamin B1 (green), α-tubulin (red) and DAPI (blue). Scale bar: 20 μm. Right panel: Asynchronous or mitotic cells were treated with H_2_O_2_ at the indicated concentrations, and the percentage of cells with abnormal nuclear shapes was measured after 10 h or 24 h. The results are shown as the means ± SDs of three independent experiments (*n* = 300). *; Control versus H_2_O_2_, ^#^; Asynchronous versus Mitosis. *P < 0.05; **^, ##^*P* < 0.01; ^###^*P* < 0.001 by Student’s *t*-test. **b** Representative examples of abnormal nuclear shapes in H_2_O_2_-treated cells. Upper panel; Lamin B1 staining (green). Lower panel: Images in the upper panels were converted to 2.5-dimensional images by using the ZEISS Microscope software ZEN. Scale bar: 5 μm. **c** Electron microscopy images of the nuclear envelope in cells treated with or without H_2_O_2_ for 10 h. Scale bar: 2.5 μm. **d** The intensity of every pixel inside an imaginary circle drawn inside a nucleus was measured, and the standard deviation of the intensities of the pixels in a single nucleus was calculated by using ZEN software. We used the same samples as in (a). The results are shown as the means ± SDs (*n* = 50). *; Control versus H_2_O_2_, ^#^; Asynchronous versus Mitosis. ^#^*P* < 0.05; **, ^##^*P* < 0.01; ^###^*P* < 0.001 by Student’s *t*-test. **e** Nuclear circularity was calculated by using ImageJ software with the same samples as in (**a**). The results are shown as the means ± SDs (*n* = 30). *; Control versus H_2_O_2_, ^#^; Asynchronous versus Mitosis. ^#^*P* < 0.05; **, ^##^*P* < 0.01; ^###^*P* < 0.001 by Student’s *t*-test

To determine whether the cue that induces abnormal nuclear shapes functions in a cell cycle-dependent manner—specifically, whether mitosis is a sensitive period for the formation of abnormal nuclear shapes—we compared the effects of H_2_O_2_ treatment on asynchronous and mitotic cells obtained following the procedure shown in Supplementary Fig. 1a. After H_2_O_2_ treatment for 10 h, which provides sufficient time for mitotic cells to enter the next interphase, during which altered nuclear morphology is observed, we monitored changes in nuclear shape by immunostaining for Lamin B1. Treatment of asynchronous cells with H_2_O_2_ had little effect on the nuclear shape in most cells except at high concentrations of H_2_O_2_ with longer treatment durations. In contrast, treatment with H_2_O_2_ during mitosis caused marked, concentration-dependent changes in nuclear shape in the subsequent interphase, inducing significant changes at an H_2_O_2_ concentration of 50 μM that plateaued at 100 μM; in both cases, nuclear shape was analyzed at 10 and 24 h after H_2_O_2_ treatment. Indeed, mitotic cells showed a significantly higher tendency than asynchronous cells to form abnormal nuclear shapes under every H_2_O_2_ treatment condition (Fig. 1a). Notably, neither 50 nor 100 μM H_2_O_2_, concentrations that are easily achievable in a pathological setting (e.g., a rat ischemia/reperfusion model^34^), caused cell death after 24 h, as we reported previously^30^.

Treatment of mitotic cells with H_2_O_2_ was followed by a variety of changes in nuclear shape, including folding or fragmentation of the nuclear envelope or adoption of a globular shape. These changes were also confirmed in simulated 3-dimensional (i.e., 2.5D) images (Fig. 1b). Furthermore, electron microscopy revealed that the nuclear envelope in cells treated with H_2_O_2_ during mitosis formed a curved section with electron-dense sites that may indicate thickening of the nuclear membrane (Fig. 1c).

To measure the abnormal nuclear shapes more objectively, we further analyzed the extent of the variability in Lamin B1 staining intensity. Since the intensity of Lamin B1 staining is more variable in folded or curved nuclei than in normally shaped nuclei, we reasoned that the standard deviation of these values would be an indicator of the degree of nuclear shape alteration. Consistent with the counts of abnormal nuclei, the Lamin B1 staining in mitotic cells exhibited a significantly larger standard deviation than that in asynchronous cells both 10 and 24 h after H_2_O_2_ treatment (Fig. 1d). The circularity of the nucleus was quantified as another approach for objectively representing changes in nuclear shape (Fig. 1e). A circularity value of “1” corresponds to a complete circle, whereas smaller values denote greater deviations from circularity. As a reference point, the mean circularity values of both control asynchronous and mitotic HeLa cells were ~0.8. Whereas the mean circularity value of asynchronous cells at 10 and 24 h after H_2_O_2_ treatment remained ~0.8 regardless of the concentration of H_2_O_2_, it was significantly reduced in mitotic cells, decreasing to ~0.7.

To determine whether the effects of H_2_O_2_ on the cell cycle were limited to continuous-exposure conditions, we also tested the effects of transient exposure to H_2_O_2_. Treatment with H_2_O_2_ for 2 h followed by wash-out produced the same susceptibility to the formation of abnormal nuclei in mitotic cells, as shown by the variability in the Lamin B1 immunostaining intensity and the circularity index (Supplementary Figs. 2a–c). This enhanced vulnerability of mitotic cells to the formation of abnormal nuclei following H_2_O_2_ treatment compared with that of asynchronous cells was observed not only in HeLa cells but also in U2OS, RPE-1 and HT1080 cells, indicating the generalizability of our observations (Supplementary Fig. 1b).

In addition, we detected changes in the immunocytochemical images of other constituents of the nuclear membrane, such as Lamin A/C, Emerin, and the NPC, in response to increases in ROS during mitosis that were associated with changes in nuclear shape (Supplementary Fig. 3). These data show that mitotic cells are prone to the formation of abnormal nuclei following oxidative stress.

### Formation of abnormal nuclei following H_2_O_2_ treatment is prevented by NAC or catalase

**Fig. 2:**
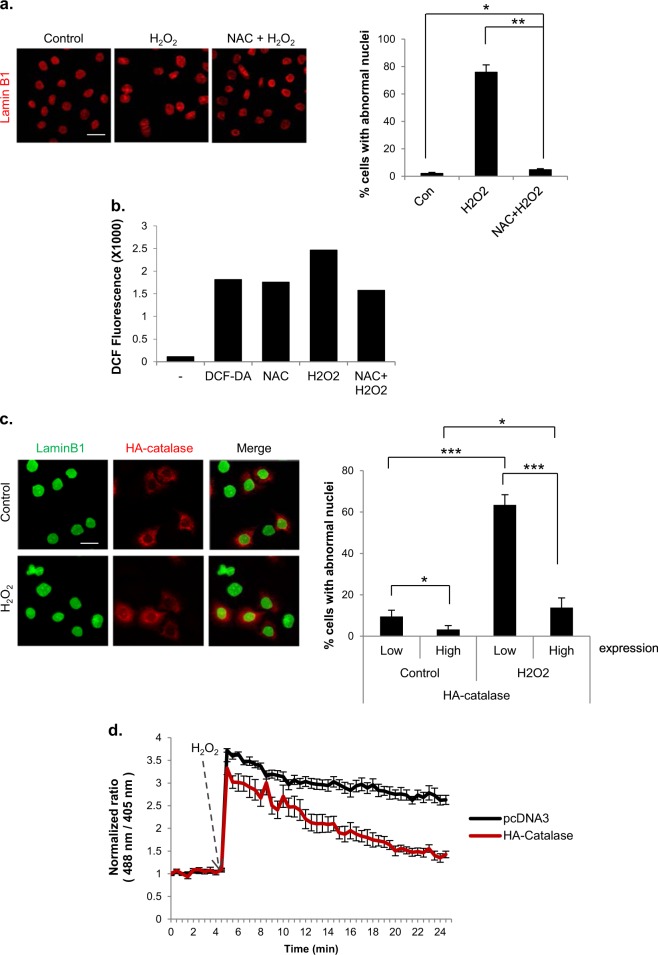
Nuclear shape alteration induced by H_2_O_2_ treatment during mitosis is rescued by antioxidants. **a** Cells were pretreated (NAC + H_2_O_2_) or not pretreated (H_2_O_2_) with NAC for 30 min. Then, mitotic cells were isolated through shake-off and treated with 100 μM H_2_O_2_ for 10 h. Left panel: After 10 h, the nuclear shape was assessed by Lamin B1 staining (red). Scale bar: 20 μm. Right panel: Percentage of cells with abnormal nuclear shapes. The results are shown as the means ± SDs of three independent experiments (*n* = 300). *P < 0.05; **P < 0.01 by Student’s *t*-test. **b** Mitotic cells were pretreated or not pretreated with NAC for 30 min and were then incubated with 100 μM H_2_O_2_ for 1 h. Intracellular ROS levels were measured by FACS analysis using DCF-DA. **c** HeLa cells were transfected with an HA-catalase expressing vector, and mitotic cells were then treated with or without 100 μM H_2_O_2_ for 10 h. Left panel: nuclear shape of HA-catalase (red)-transfected cells. Scale bar: 20 μm. Right panel: percentage of cells with abnormal nuclear shapes according to HA-catalase expression. The results are shown as the means ± SDs of three independent experiments (*n* = 100). *P < 0.05; **P < 0.01; ***P < 0.001 by Student’s *t*-test. **d** HeLa cells were transfected with pHyper-Cyto and then with pcDNA3 or HA-Catalase. Changes in intracellular H_2_O_2_ levels are expressed as the fluorescence intensity ratios upon excitation with 488 and 405-nm lasers. Representative time-lapse fluorescence images (30 s intervals for 25 min) of pHyper-cyto sensors in mitotic HeLa cells treated with 100 μM H_2_O_2_

To confirm that the formation of abnormal nuclear shapes in H_2_O_2_-treated mitotic cells was actually caused by ROS, we pretreated cells with the antioxidant N-acetyl-L-cysteine (NAC). Indeed, changes in nuclear shape after H_2_O_2_ treatment were almost completely prevented by NAC (Fig. 2a). The ROS-lowering effect of NAC on mitotic cells under these treatment conditions was verified by fluorescence-activated cell sorting (FACS) analysis using the fluorescent ROS indicator dichlorodihydrofluorescein diacetate (DCF-DA) (Fig. 2b).

To more specifically address whether H_2_O_2_ was responsible for the formation of abnormal nuclei, we transfected cells with catalase, an enzyme that converts H_2_O_2_ to water and oxygen, prior to H_2_O_2_ treatment. Whereas cells that did not express catalase exhibited a change in nuclear shape, as expected, the nuclear shape change was markedly reduced in cells overexpressing catalase (Fig. 2c). Interestingly, the formation of abnormal nuclei was also suppressed in catalase-expressing cells in the absence of H_2_O_2_ treatment, suggesting that basal levels of H_2_O_2_ induce the formation of a basal level of abnormal nuclei. The H_2_O_2_-lowering effect of ectopically expressed catalase was confirmed using pHyper-Cyto (Fig. 2d), a specific fluorescent protein probe for H_2_O_2_^32^. Collectively, these findings indicate that mitotic cells are more prone to the formation of abnormal nuclei following H_2_O_2_ treatment than asynchronous cells and that this phenomenon is directly attributable to ROS, based on the preventive effect of NAC treatment and catalase overexpression.

### Neither quantitative changes in Lamin B1 levels nor DNA damage are major contributors to H_2_O_2_-induced nuclear shape changes

**Fig. 3:**
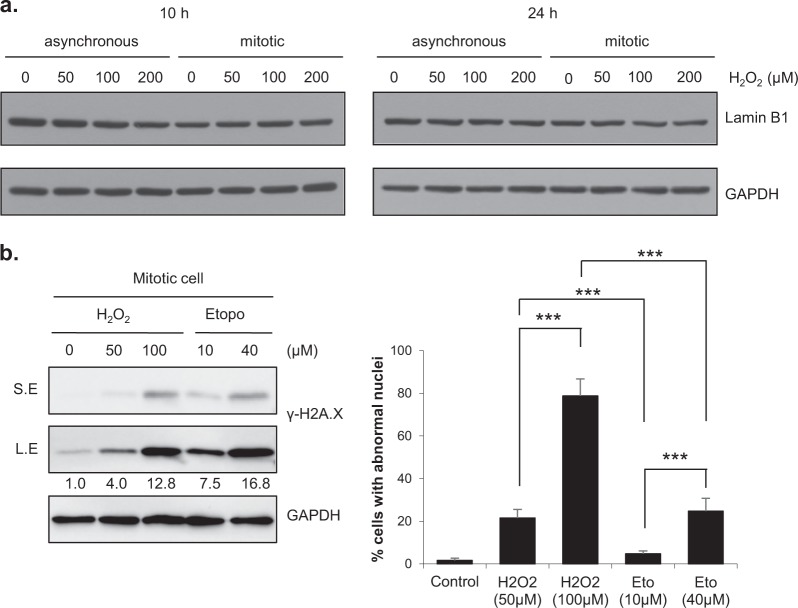
H_2_O_2_-induced abnormal nuclear shapes are mainly due neither to changes in the Lamin B1 level nor to DNA damage. **a** Asynchronous or mitotic HeLa cells were treated with H_2_O_2_ at the indicated concentrations for 10 h or 24 h. Cell lysates were harvested and subjected to western blot analysis by using the indicated antibodies. **b** Mitotic HeLa cells were treated with H_2_O_2_ or etoposide at the indicated concentrations. Left panel: Mitotic cells were cotreated with H_2_O_2_ or etoposide and 100 ng/ml nocodazole for 1 h and subjected to western blot analysis with the indicated antibodies. Right panel: Mitotic HeLa cells were treated with H_2_O_2_ or etoposide for 10 h, and the percentage of cells with abnormal nuclear shapes was then determined. The results are shown as the means ± SDs of three independent experiments (*n* = 300). ***P < 0.001 by Student’s *t*-test. *S.E.* short exposure; *L.E.* long exposure

Previous studies have reported that p38 MAPK (mitogen-activated protein kinase) is activated by ROS in ataxia-telangiectasia cells and that the level of endogenous Lamin B1 is increased in these cells, resulting in nuclear deformation and senescence^13^. Therefore, we investigated whether the formation of abnormal nuclei in response to H_2_O_2_ exposure under our experimental conditions was accompanied by changes in the level of Lamin B1. Lamin B1 levels were not noticeably changed at 10 or 24 h after treatment with H_2_O_2_ (Fig. 3a). Similar results were obtained when the cells were transiently treated with H_2_O_2_ (Supplementary Fig. 2d), excluding the possibility that quantitative changes in Lamin B1 levels are involved in H_2_O_2_-induced formation of abnormal nuclei.

Oxidative stress is a well-known cause of DNA damage^35,36^, and we previously reported that H_2_O_2_ induces DNA damage and subsequent chromatin bridge formation in mitotic cells, changes that appear to be related to binucleation^30^. To determine whether DNA damage is involved in the formation of abnormal nuclei, we compared the effects of H_2_O_2_ and etoposide, a topoisomerase II inhibitor that induces DNA double-strand breaks. Notably, treatment with a high concentration of etoposide induced an increase in the number of cells with abnormal nuclei, suggesting that DNA damage does contribute to the formation of abnormal nuclei. However, although the expression of the DNA damage marker γ-H2A.X was increased to a greater extent by 10 μM etoposide than by 50 μM H_2_O_2_, the percentage of cells that formed abnormal nuclei was significantly lower in response to 10 μM etoposide than in response to 50 μM H_2_O_2_. A comparison of treatment with 40 μM etoposide and 100 μM H_2_O_2_ showed the same tendency (Fig. 3b), indicating that DNA damage plays at most a modest role in the formation of abnormal nuclei in our system. Therefore, mechanisms other than DNA damage appear to be of primary importance in the nuclear shape changes induced by H_2_O_2_.

### H_2_O_2_ inhibits PP2A activity during mitosis

**Fig. 4:**
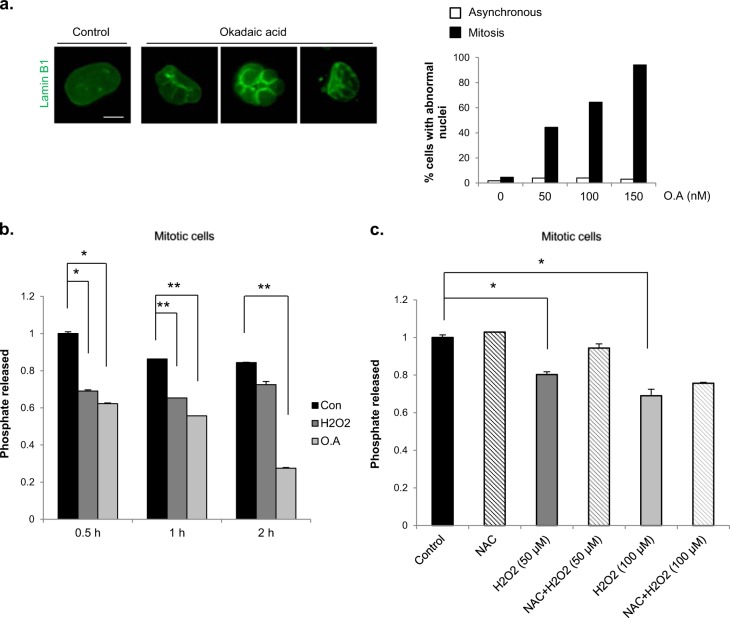
Inhibition of protein phosphatase 2 A activity is involved in H_2_O_2_-induced abnormal nuclei formation. **a** Mitotic HeLa cells were treated with okadaic acid in a dose-dependent manner for 2 h. Cells were washed, and nuclear shape alterations were observed after 8 h. Left panel: Representative examples of the nuclear shape change in cells treated with okadaic acid. Scale bar: 10 μm. Right panel: Percentage of cells with abnormal nuclear shapes resulting from treatment with okadaic acid (O.A) at the indicated concentrations (*n* = 100). **b** Mitotic HeLa cells were treated with 100 μM H_2_O_2_ or 100 nM okadaic acid (O.A) for the indicated durations. The cells were harvested, and PP2A activity was determined using the PP2A activity assay kit from R&D Systems. The results are shown as the means ± SDs of three independent experiments. *P < 0.05; **P < 0.01 by Student’s *t*-test. **c** Cells were pretreated (NAC + H_2_O_2_) or not pretreated (H_2_O_2_) with NAC for 30 min before H_2_O_2_ treatment. Mitotic cells were isolated through shake-off and treated with or without H_2_O_2_ for 30 min. The cells were harvested, and PP2A activity was determined using the PP2A activity assay kit. The results are shown as the means ± SDs of three independent experiments. *P < 0.05 by Student’s *t*-test

Because nuclear envelope disassembly and reassembly occur during mitotic entry and exit, respectively, we hypothesized that the observed propensity for mitotic cells to undergo changes in nuclear shape in response to H_2_O_2_ was attributable to the effects of H_2_O_2_ on nuclear envelope disassembly and/or reassembly processes. It has previously been shown that PP2A plays an important role in the nuclear envelope reassembly process during mitotic exit^16^. To determine whether PP2A is involved in the formation of abnormal nuclei in our experimental system, we investigated changes in nuclear shape following the treatment of mitotic cells with different concentrations of the PP2A inhibitor okadaic acid. Although both PP1 and PP2A are inhibited by okadaic acid, it has been reported that PP2A is more sensitive to okadaic acid (in vitro IC_50_≈0.5 nM) than PP1 (IC_50_≈42 nM)^18,37^. Treatment of mitotic cells with okadaic acid for 2 h caused robust, concentration-dependent changes in nuclear shape, affecting ~94 % of cells at the highest concentration tested (150 nm); by contrast, okadaic acid had little effect on nuclear shape in asynchronous cells at any concentration (Fig. 4a). Thus, PP2A inhibition results in the formation of abnormal nuclei, but only when inhibition occurs during mitosis, a phenomenon comparable to the observed greater vulnerability of mitotic cells than asynchronous cells to H_2_O_2_-induced nuclear shape changes. Since H_2_O_2_ is known to decrease PP2A activity in asynchronous cells^17–19^, we investigated whether PP2A activity was also reduced by H_2_O_2_ in mitotic cells (Fig. 4b). In vitro PP2A activity was assayed after the treatment of mitotic cells with H_2_O_2_ or okadaic acid for different durations. Indeed, both H_2_O_2_ and okadaic acid decreased the activity of PP2A in mitotic cells. In addition, the H_2_O_2_-induced decrease in PP2A activity was found to be dependent on the H_2_O_2_ concentration and showed a tendency towards recovery in cells treated with NAC (Fig. 4c). Therefore, H_2_O_2_ inhibits the activity of PP2A in mitotic cells, potentially affecting the nuclear envelope reassembly process and causing changes in nuclear shape.

### Ectopic expression of PP2A rescues H_2_O_2_-induced aberrant nuclear shape changes

**Fig. 5:**
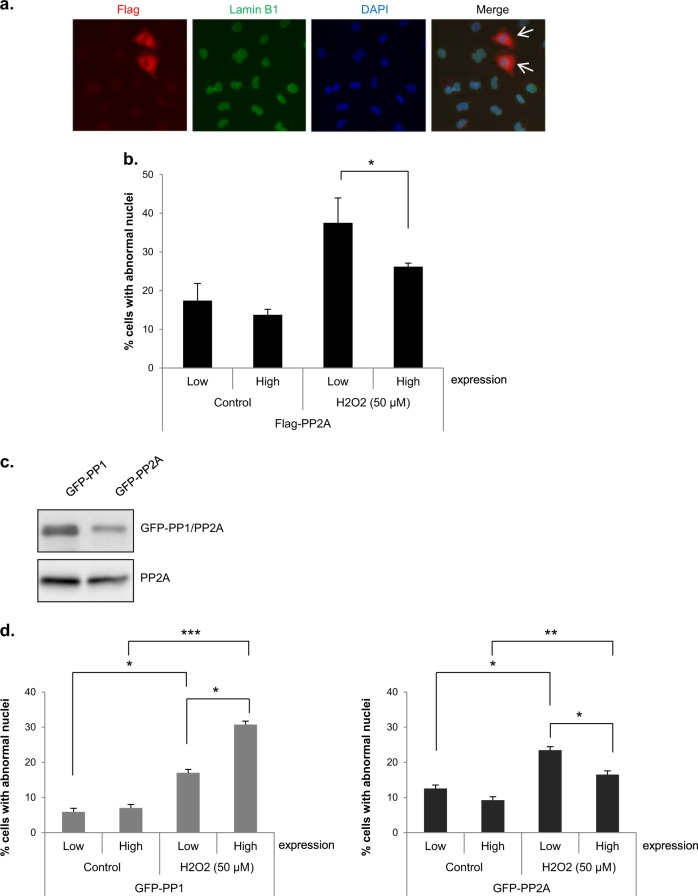
Overexpression of PP2A but not PP1 rescued the H_2_O_2_-induced nuclear shape alteration. **a**, **b** HeLa cells were transfected with a Flag-PP2A expression vector, and mitotic cells were then treated with 50 μM H_2_O_2_ for 10 h. Representative image of mitotic cells expressing Flag-PP2A (red, arrow) after treatment with H_2_O_2_ for 10 h. Lamin B1 (green) and DAPI (blue) (**a**). Percentage of cells with abnormal nuclear shapes according to Flag-PP2A expression. The results are shown as the means ± SDs of three independent experiments (*n* = 100). *P < 0.05 by Student’s *t*-test (**b**). **c**, **d** HeLa cells were separately transfected with GFP-PP1 or GFP-PP2A expression vectors and mitotic cells were then treated with 50 μM H_2_O_2_. Mitotic cell lysates were harvested and subjected to western blot analysis with an anti-GFP antibody (for PP1 or PP2A expression). PP2A; loading control (**c**). 10 h after H_2_O_2_ treatment, the percentage of cells with abnormal nuclei according to GFP-PP1 or GFP-PP2A expression was determined. The results are shown as the means ± SDs of three independent experiments (*n* = 100). *P < 0.05; **P < 0.01; ***P < 0.001 by Student’s *t*-test (**d**)

To verify the relationship between the decrease in PP2A activity and the abnormal nuclei formation, we investigated whether ectopic expression of PP2A rescued H_2_O_2_-induced nuclear shape changes. After overexpression of a Flag-tagged PP2A catalytic subunit, mitotic cells were collected and treated with 50 μM H_2_O_2_ to induce changes in nuclear shape. In the absence of H_2_O_2_ treatment, exogenously expressed PP2A had no effect on nuclear shape (~17% and 14% cells with aberrant nuclei with low- and high-level PP2A expression, respectively). In contrast, overexpression of PP2A partially abrogated H_2_O_2_-induced abnormal nuclei formation; the treatment of mitotic cells with 50 μM H_2_O_2_ induced nuclear shape changes in 37 and 26% of cells expressing low and high levels of PP2A, respectively (Figs. 5a, b), strongly indicating that the decrease in PP2A activity induced by H_2_O_2_ is involved in H_2_O_2_-induced abnormal nuclei formation.

Since PP1 is inhibited by H_2_O_2_ in vitro^38^ and is an important phosphatase during mitosis^3,14,15,39^, we ectopically overexpressed GFP-tagged PP1 and PP2A and compared the effects of these phosphatases overexpression on nuclear shape alterations (Figs. 5c, d). PP2A-overexpressing cells again showed a reduction in H_2_O_2_-induced nuclear shape changes, but PP1-overexpressing cells did not, instead exhibiting an increase that remains unexplained. These results indicate that PP2A but not PP1 plays an important role in the reassembly of the nuclear envelope during mitosis and that inhibition of PP2A activity by H_2_O_2_ causes nuclear deformation.

### Inhibition of PP2A activity by H_2_O_2_ causes the mislocalization of core proteins during mitotic exit

**Fig. 6:**
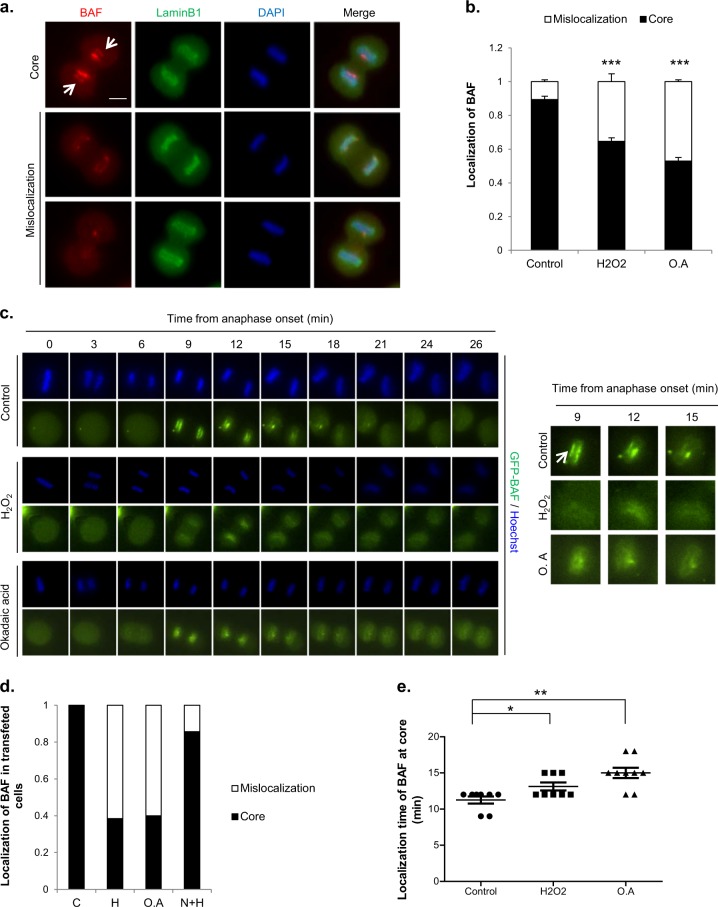

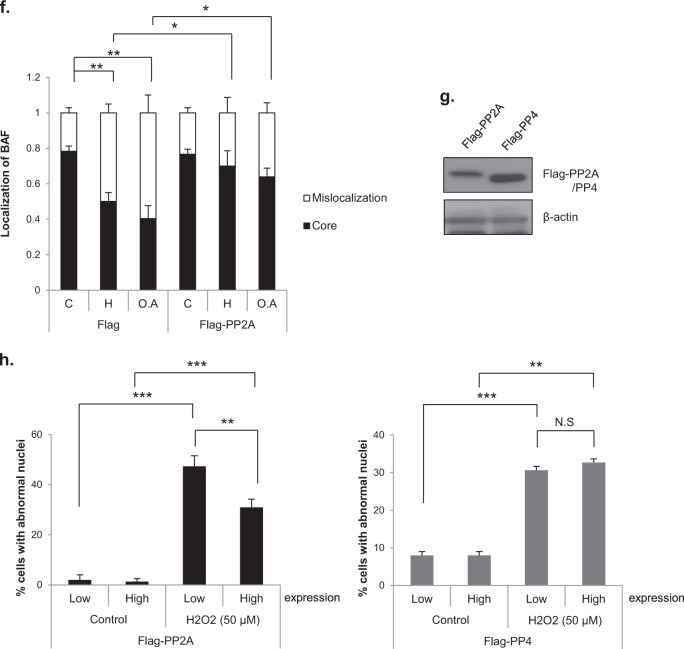
Inhibition of PP2A by H_2_O_2_ influences BAF dynamics and nuclear envelope formation. **a**, **b** Mitotic HeLa cells were treated with 50 μM H_2_O_2_ or 100 nM okadaic acid (O.A) for 30 min. Then, the cells were immunostained for endogenous BAF (red), Lamin B1 (green) and DAPI (blue). BAF localization in telophase was classified into two categories: localization at the “core” region (upper, arrow), observed in control cells, and mislocalization, which means either that BAF is located in both ‘core’ and “noncore” regions (middle) or that the BAF signal is rather absent (lower). Scale bar: 10 μm (**a**). Quantification of BAF localization. The results are shown as the means ± SDs of three independent experiments (*n* = 50). ***P < 0.001 by Student’s t-test, compared to the control (**b**). **c** Time-lapse imaging of GFP-BAF. HeLa cells were transfected with a GFP-BAF expression vector and seeded in 4-well plates. Cells were synchronized at the G2 phase by using RO3306, a Cdk1 inhibitor, and then released from arrest and stained with Hoechst 33342 to visualize chromosomes during the time-lapse analysis. After 30 min, cells were treated with 50 μM H_2_O_2_ or 100 nM okadaic acid and observed at 3-min intervals. Left panel; time-lapse images, with time 0 indicating anaphase onset. Right panel; Magnified images of the left panel. The arrow indicates BAF localization at the “core” region. **d** Quantitation GFP-BAF localization from the time-lapse analysis. The results are shown as the means (*n* = 15). **e** Quantitation of the localization time of GFP-BAF at the core region from the time-lapse analysis. The results are shown as the means ± SDs (*n* = 8). **f** HeLa cells were transfected with mock (Flag) or Flag-PP2A expression vectors, and mitotic cells were then treated with 50 μM H_2_O_2_ (H) or 100 nM okadaic acid (O.A). BAF localization was analyzed in telophase cells. C; control. The results are shown as the means ± SDs of three independent experiments (*n* = 50). *P < 0.05; **P < 0.01 by Student’s *t*-test. **g** and **h** HeLa cells were separately transfected with Flag-PP2A or Flag-PP4 expression vectors, and mitotic cells were then treated with 50 μM H_2_O_2_. Mitotic cell lysates were harvested and subjected to western blot analysis with an anti-Flag antibody (for PP2A or PP4 expression). β-Actin; loading control (**g**). 10 h after H_2_O_2_ treatment, the percentage of cells with abnormal nuclei according to Flag-PP2A or Flag-PP4 expression was determined. The results are shown as the means ± SDs of three independent experiments (*n* = 100). *P < 0.05; **P < 0.01; ***P < 0.001 by Student’s *t*-test (**h**)

PP2A dephosphorylates BAF, thereby enabling BAF to localize to the core region during the nuclear envelope reassembly process^16,24^. We investigated whether H_2_O_2_ treatment induced changes in the phosphorylation status of BAF. BAF was seen as two bands in the western blots, and the upper band was confirmed through lambda phosphatase treatment to be the phosphorylated form of BAF. In the control group, immediately after shake-off, BAF was mostly phosphorylated, and BAF phosphorylation was almost abolished by 30 min after shake-off. However, in the H_2_O_2_-treated cells, the disappearance of phosphorylation happened as late as 90 min after shake-off. Therefore, BAF phosphorylation persisted longer after treatment with H_2_O_2_ (Supplementary Fig. 4). To determine whether H_2_O_2_-induced decreases in PP2A activity affect BAF localization, we monitored BAF localization during telophase after the treatment of mitotic cells with 50 µM H_2_O_2_ or 100 nM okadaic acid. BAF localization was assessed only in early telophase cells, in which chromosome decondensation had not occurred but the formation of the nuclear envelope around the set of chromosomes near the spindle pole had been initiated. We classified BAF localization during telophase into two categories: normal localization in the core and mislocalization, meaning that BAF was either distributed between “core” and “noncore” regions or was undetectable (Fig. 6a). Whereas BAF was localized to the core region in most control cells, it was poorly localized to the core region in cells treated with H_2_O_2_ or okadaic acid (Fig. 6b).

We further performed time-lapse analyses using GFP-BAF–overexpressing cells. These experiments also revealed that the localization of BAF at the core region was robustly decreased by H_2_O_2_ or okadaic acid (Figs. 6c, d). Moreover, even in cells showing BAF localization to the core region, BAF recruitment was significantly delayed in cells treated with H_2_O_2_ or okadaic acid (Fig. 6e). We also addressed whether the mislocalization of BAF could be rescued by enhancing PP2A activity through overexpression of the Flag-tagged PP2A catalytic subunit. Indeed, appropriate localization of BAF was restored in H_2_O_2_- or okadaic acid-treated cells by overexpression of Flag-PP2A (Fig. 6f), indicating that inhibition of PP2A by H_2_O_2_ or okadaic acid induced BAF mislocalization, a phenomenon that paralleled abnormal nuclei formation. Given that both knocking down BAF and perturbing the phosphorylation status of BAF by knocking down VRK1, a kinase that phosphorylates BAF during mitosis, have been reported to induce abnormal nuclear shapes^27–29,40^, it is likely that BAF mislocalization induced by H_2_O_2_ or okadaic acid causes the abnormal nuclear shapes.

To further confirm that changes in the dephosphorylation of BAF by H_2_O_2_ are involved in the formation of abnormal nuclei, we constructed a phospho-dead BAF mutant. Since BAF is phosphorylated by VRK1, for which Ser 4 is known as the major phosphorylation site during mitotic entry, we constructed an S4A mutant^41,42^. Consistent with a previous report^40^, whereas GFP-BAF WT did not return to the chromosome until anaphase, the S4A mutant was continuously localized to the chromosome during mitosis (Supplementary Fig. 5a). Endogenous BAF was depleted using 3′-UTR-targeting siBAF, and GFP-BAF WT or S4A was overexpressed (Supplementary Fig. 5b). Then, mitotic cells were treated with H_2_O_2_, and the nuclear shape changes after 10 h were assessed (Supplementary Fig. 5c). Unfortunately, consistent with the findings of a previous report^40^, nuclear shape changes were induced by expression of the BAF S4A mutant even without H_2_O_2_ treatment, possibly via the effect of this mutation on nuclear envelope disassembly (lower left panel). To compensate for the effect of expression of the S4A mutant on the formation of abnormal nuclei without H_2_O_2_, we measured the ratio of the abnormal nuclei in the H_2_O_2_ treatment group to the abnormal nuclei in the control nontreated group to determine the degree of abnormal nuclei formation induced by H_2_O_2_ treatment but not by expression of the S4A mutant (lower right panel). The results showed that overexpression of the phospho-dead BAF mutant partially abrogated H_2_O_2_-induced abnormal nuclei formation, suggesting that defects in BAF dephosphorylation at Ser 4 resulting from H_2_O_2_ treatment induced the nuclear shape changes.

We also investigated whether the localization of Lamin A, another core protein recruited to the core by BAF^25^, is associated with BAF mislocalization (Supplementary Fig. 6). Indeed, the mislocalization of BAF was significantly correlated with the mislocalization of Lamin A (Table 1-1). This positive correlation between the localization of BAF and the localization of Lamin A was also observed in cells treated with H_2_O_2_ or okadaic acid (Table 1-2,3), indicating that the mislocalization of BAF hinders the proper localization of other core proteins. It is quite possible that the mislocalization of key core proteins, including Lamin A/C, induces abnormal reassembly of the nuclear envelope and subsequent abnormalities in nuclear shape.

**Table 1:**
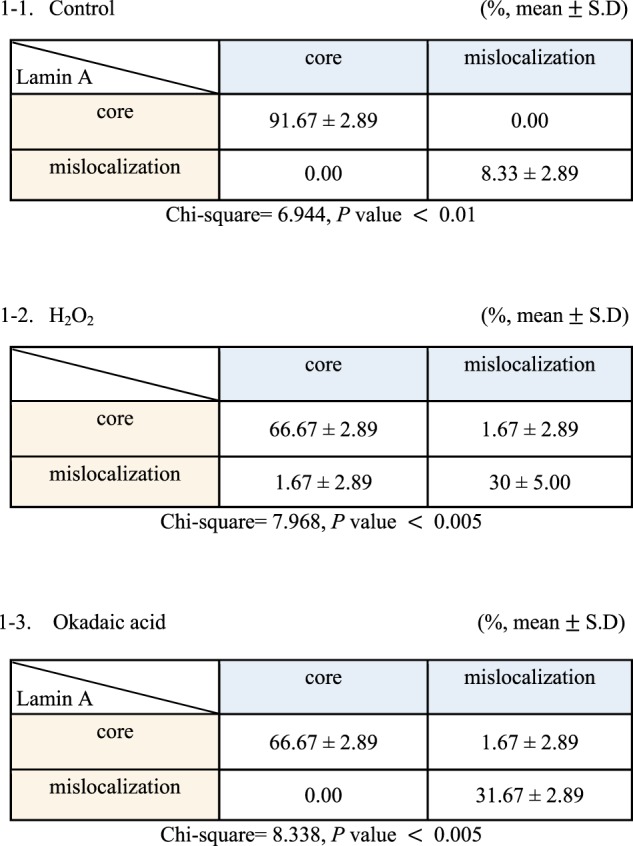
Localization of Lamin A/C by BAF localization in the telophase chromosome core region

It has recently been reported that BAF is dephosphorylated by another phosphatase, PP4, during mitotic telophase^37^. To determine whether PP4 is also involved in H_2_O_2_-induced nuclear shape changes, we overexpressed Flag-PP2A or Flag-PP4 and compared the ability of these phosphatases to rescue nuclear shape changes induced by H_2_O_2_ (Figs. 6g, h). In our experimental system, overexpression of PP2A but not PP4 was able to rescue nuclear shape changes induced by H_2_O_2_ treatment. Collectively, our data indicate that, among the phosphatases PP1, PP2A and PP4, which are known to be involved in mitosis^43^, PP2A is likely the phosphatase involved in H_2_O_2_-induced nuclear shape changes.

Taken together, our findings support the conclusion that the H_2_O_2_-induced decrease in PP2A activity during mitosis leads to the mislocalization of BAF and Lamin A/C during nuclear envelope reassembly, ultimately resulting in an abnormal nuclear shape.

## Discussion

Recent studies have revealed a variety of mechanisms that lead to nuclear shape changes. For example, defects in lamin and inner and outer nuclear membrane proteins, such as Lem4, Lap2β and LBR^16,44–47^, and defects in proteins that affect cytoskeletal tension, such as the LINC (linker of nucleoskeleton and cytoskeleton) complex, actin and tubulin^44,48,49^, lead to changes in nuclear shape. In addition, defects in BAF, a core protein that connects the nuclear envelope and chromatin, induce nuclear deformation^27–29^, reflecting defects in other core proteins (e.g., Emerin, Lap2β, and Lamin A)^44^. VRK1 is the protein kinase of BAF that phosphorylates Ser 4 on BAF during mitotic entry, and a defect in VRK1, in turn, causes BAF to remain on mitotic chromosomes, thus increasing anaphase bridges and multipolar spindles, ultimately disrupting the morphology of the nuclear envelope^40^. Moreover, transcription factors (e.g., GATA6) and chromatin remodeling factors (e.g., BRG1) that are not directly involved in regulating the structure of the nuclear envelope can induce changes in nuclear shape by reducing the expression of proteins such as Emerin^50,51^.

**Fig. 7:**
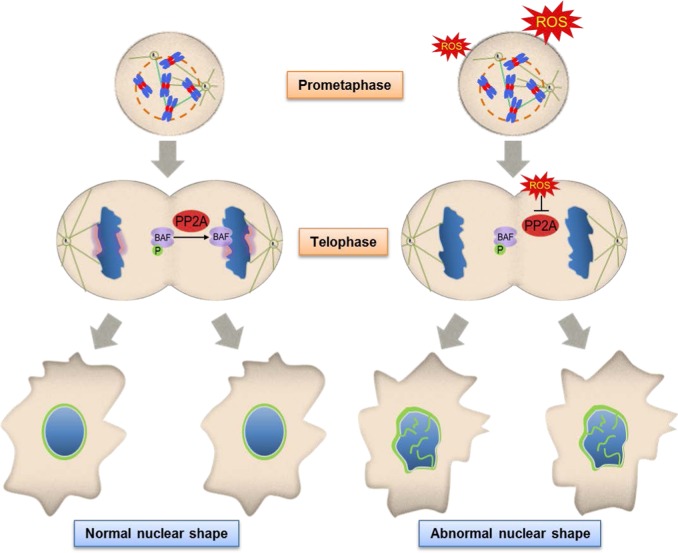
Inhibition of PP2A activity by ROS during mitosis results in abnormal nuclear shapes via the mislocalization of BAF, a substrate of PP2A, during nuclear envelope reassembly. Schematic showing how the increase in H_2_O_2_ during mitosis induces nuclear shape alterations. BAF localizes to the chromosomal core region during telophase through dephosphorylation by PP2A, and the constituents of other nuclear membranes are subsequently recruited around the chromosome to form a normal nuclear shape. However, increasing H_2_O_2_ in mitotic cells induces the inhibition of PP2A activity. A decrease in PP2A activity seems to prevent BAF from being localized in the chromosome core region during telophase, which subsequently mislocalizes other proteins (e.g., Lamin A) that enter the core region in a BAF-dependent manner, thus resulting in the formation of an abnormal nuclear shape

Most of the above mentioned studies have shown that the formation of abnormal nuclei is a cell cycle-independent event induced by the deletion/mutation of specific proteins. Even in the case of ataxia-telangiectasia cells, ROS induce nuclear shape changes by altering the amount of Lamin B1 protein^13^. Here, we suggest that pathophysiologically achievable concentrations of H_2_O_2_ affect nuclear envelope reassembly by decreasing the activity of PP2A and causing the subsequent mislocalization of its substrate, BAF, from its normal core position (Fig. 7). BAF mislocalization appears to affect the proper localization of Lamin A/C, another well-known core protein (Table 1), suggesting the possibility that the recruitment of other core proteins involved in the nuclear envelope reassembly process is also prevented, resulting in an abnormal nuclear envelope reassembly process and a malformed nucleus (Fig. 7). Our model of abnormal nuclei formation suggests the interesting possibility that environmental cues such as ROS can efficiently induce changes in nuclear shape by altering the function and/or intracellular localization of certain proteins and that these environmental cues affect cells in a specific stage of the cell cycle because they target the nuclear envelope reassembly process, which occurs only during mitosis. Since it is well known that ROS are involved in many pathological conditions, including cancer, their capacity to induce nuclear shape changes might provide novel insights into their role in these pathological conditions.

BAF is recruited to the core region via microtubules during early telophase and provides a platform for LEM-domain proteins^21,25,26^. In addition to LEM-domain proteins, other proteins, such as SUN2 and A-type Lamin, localize at the core region in a BAF-dependent manner; these proteins are collectively called ‘core’ proteins^25,26^. Consistent with this process, a BAF mutant (BAF-G25E) that does not localize to the core region was shown to be unable to recruit Emerin, Lap2β, or Lamin A to the core region during nuclear envelope reassembly; thus, these proteins remained in the cytosol during the next interphase^22^. In addition, overexpression of a Lap2β truncation mutant has been reported to change lamina assembly and nuclear envelope formation in Xenopus oocyte extracts^44,52^. Therefore, BAF-mediated recruitment of core proteins to their correct positions would appear to have a clear effect on nuclear morphology. Our observation that the localization of BAF and Lamin A/C was altered by H_2_O_2_ treatment during mitosis and was rescued by PP2A overexpression (Fig. 6, Table 1) strongly supports the conclusion that H_2_O_2_ induces abnormal nuclear shapes through its inhibitory effect on PP2A and the subsequent mislocalization of BAF and other core proteins (e.g., Lamin A).

What are the consequences of changes in nuclear shape? It has been shown that structural defects in the nuclear envelope in ovarian cancer cells directly lead to chromosomal numerical instability and aneuploidy^9^. In addition, several studies have shown that the NPC, a component of the nuclear envelope, is closely related to genome integrity^53,54^. Given that the numerical instability of chromosomes as well as derangements caused by genomic instability are widely accepted causes of tumorigenesis and tumor progression^55,56^, abnormalities in nuclear shape or the NPC might contribute to tumor formation and/or tumor progression. Our observation that normal cells (RPE1) as well as various cancer cells (HeLa, U2OS, and HT1080) showed nuclear shape changes in response to H_2_O_2_ exposure during mitosis (Supplementary Fig. 1b) strengthens these inferences. The nuclear envelope has been reported to regulate gene expression through interactions with transcription factors as well as effects on chromatin organization^57,58^. We found that H_2_O_2_ treatment caused the aggregation of NPC subunits (Supplementary Fig. 3b). In addition, electron microscopy images revealed an electron-dense region in nuclei with abnormal shapes (Fig. 1c). In this region, the NPC might also be different, and both genomic stability and gene expression are expected to be different between these cells and normal cells, a possibility that warrants further investigation.

A missense mutation (A12T) in BAF has been reported to result in a decrease in BAF protein levels and an increase in nuclear envelope abnormalities in association with a premature aging syndrome called Néstor–Guillermo progeria syndrome^29^. Moreover, Lamin A mutations cause rare clinical disorders called laminopathies, including autosomal Emery–Dreifuss muscular dystrophy and Hutchinson–Gilford progeria syndrome^59^, which are accompanied by abnormalities in nuclear morphology. Whether and how an abnormal nuclear shape causes the pathologies of these diseases warrant further investigation.

Many existing anticancer therapeutics, as well as those under development, are antimitotic agents. However, cancer cells often adapt to these drugs, resulting in mitotic ‘slippage’ and the subsequent survival of cancer cells; these drugs are also cytotoxic to normal dividing cells^60,61^. With the emergence of these side effects, new studies have been conducted to identify new cancer cell-specific drugs^62^. To specifically kill cancer cells, it is necessary to identify and target cellular characteristics unique to cancer cells, such as nuclear deformation. Therefore, investigating the phenomenon of nuclear shape changes—one of the defining characteristics of cancer cells—might foster the development of future anticancer therapies.

## Supplementary information

Supplementary Figures
