# Supplementary material for: Inhibition of PP2A activity by H2O2 during mitosis disrupts nuclear envelope reassembly and alters nuclear shape
Source: Exp Mol Med. 2019 Jun 5;51(6):64. doi: 10.1038/s12276-019-0260-0 (PMC6548778; doi:10.1038/s12276-019-0260-0)
Supplement: Supplementary file 1 — Supplementary Figures [file 12276_2019_260_MOESM1_ESM.pdf]

a.

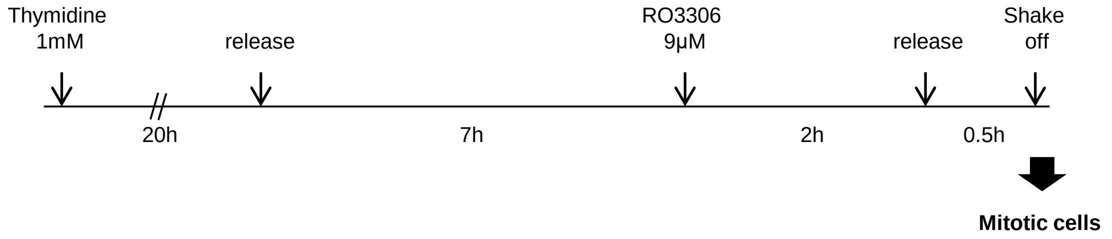

b.

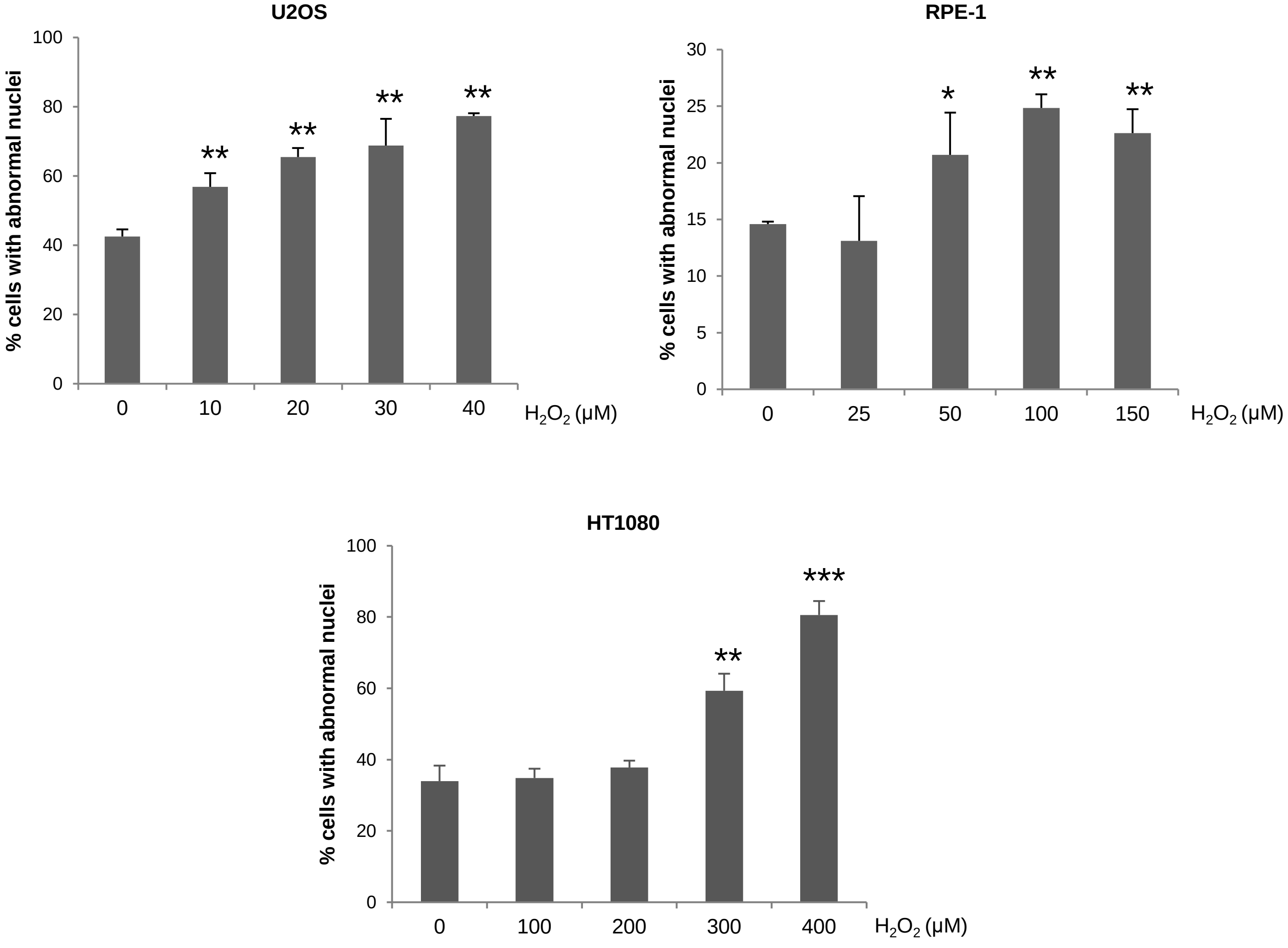

Supplementary Figure 1. H_2O_2 treatment to mitotic cells induces abnormal nuclear shape. (a) Experimental design to obtain mitotic cells. (b) Mitotic U2OS, RPE-1 and HT1080 cells were treated with H_2O_2 at indicated concentrations, and percentage of cells with abnormal nuclear shape was determined after 10 h. Results are shown as the mean ± SD from three independent experiments (n=300), *p<0.05, **p<0.01, ***p<0.001 by Student's t-test.

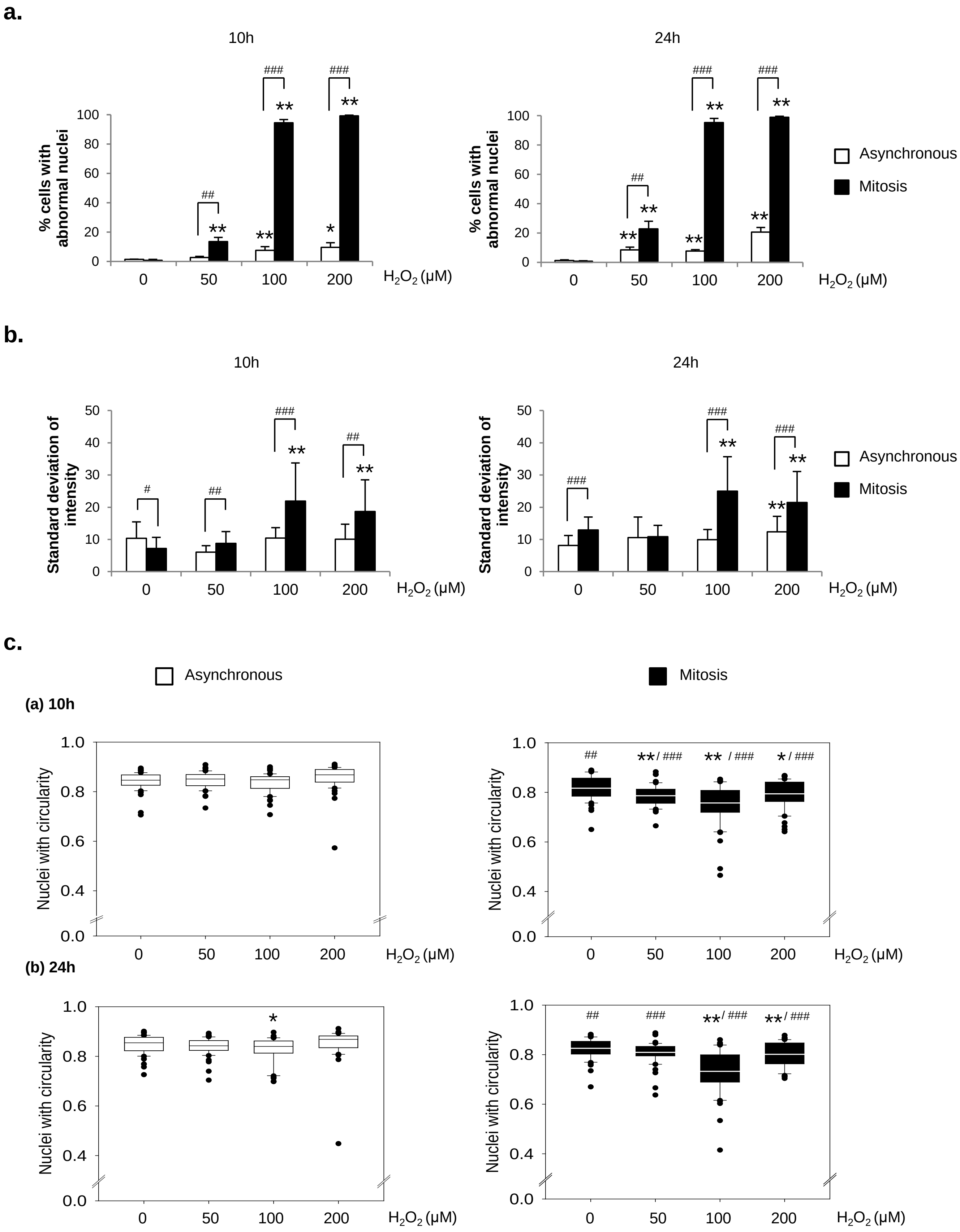
a.
10h
24h
% cells with abnormal nuclei
Asynchronous
Mitosis
H2O2 (μM)
0
50
100
200
b.
10h
24h
Standard deviation of intensity
Asynchronous
Mitosis
H2O2 (μM)
c.
Asynchronous
Mitosis
(a) 10h
(b) 24h
Nuclei with circularity
H2O2 (μM)

d.

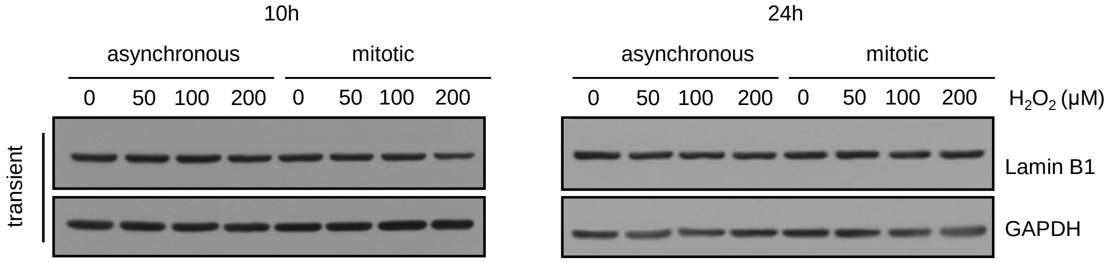

Supplementary Figure 2. Transient H_2O_2 treatment to mitotic cells induces more abnormal nuclear shape than asynchronous cells. (a) Asynchronous or mitotic cells were treated with H_2O_2 at indicated concentrations. Two hours later, cells were washed out, and the percentage of cells with abnormal nuclear shape was determined 10 h or 24 h after H_2O_2 treatment. Results are shown as the mean ± SD from three independent experiments (n=300), *; Control versus H_2O_2, $^{\#}$; Asynchronous versus Mitosis, *p<0.05, **, $^{\#\#}$p<0.01, $^{\#\#\#}$p<0.001 by Student's t-test. (b) Standard deviation of Lamin B1 intensity inside an imaginary circle in the nucleus was measured by using ZEN software with the same samples in (a). Results are shown as the mean ± SD (n=50), *; Control versus H_2O_2, $^{\#}$; Asynchronous versus Mitosis, $^{\#}$p<0.05, **, $^{\#\#}$p<0.01, $^{\#\#\#}$p<0.001 by Student's t-test. (c) Nuclear circularity was analyzed with ImageJ with the same samples in (a). Results are shown as the mean (n=30). *; Control versus H_2O_2, $^{\#}$; Asynchronous versus Mitosis, *p<0.05, **, $^{\#\#}$p<0.01, $^{\#\#\#}$p<0.001 by Student's t-test. (d) Asynchronous or mitotic HeLa cells were obtained as described in (a). Cell lysates were harvested and subjected to western blot analysis by using indicated antibodies.

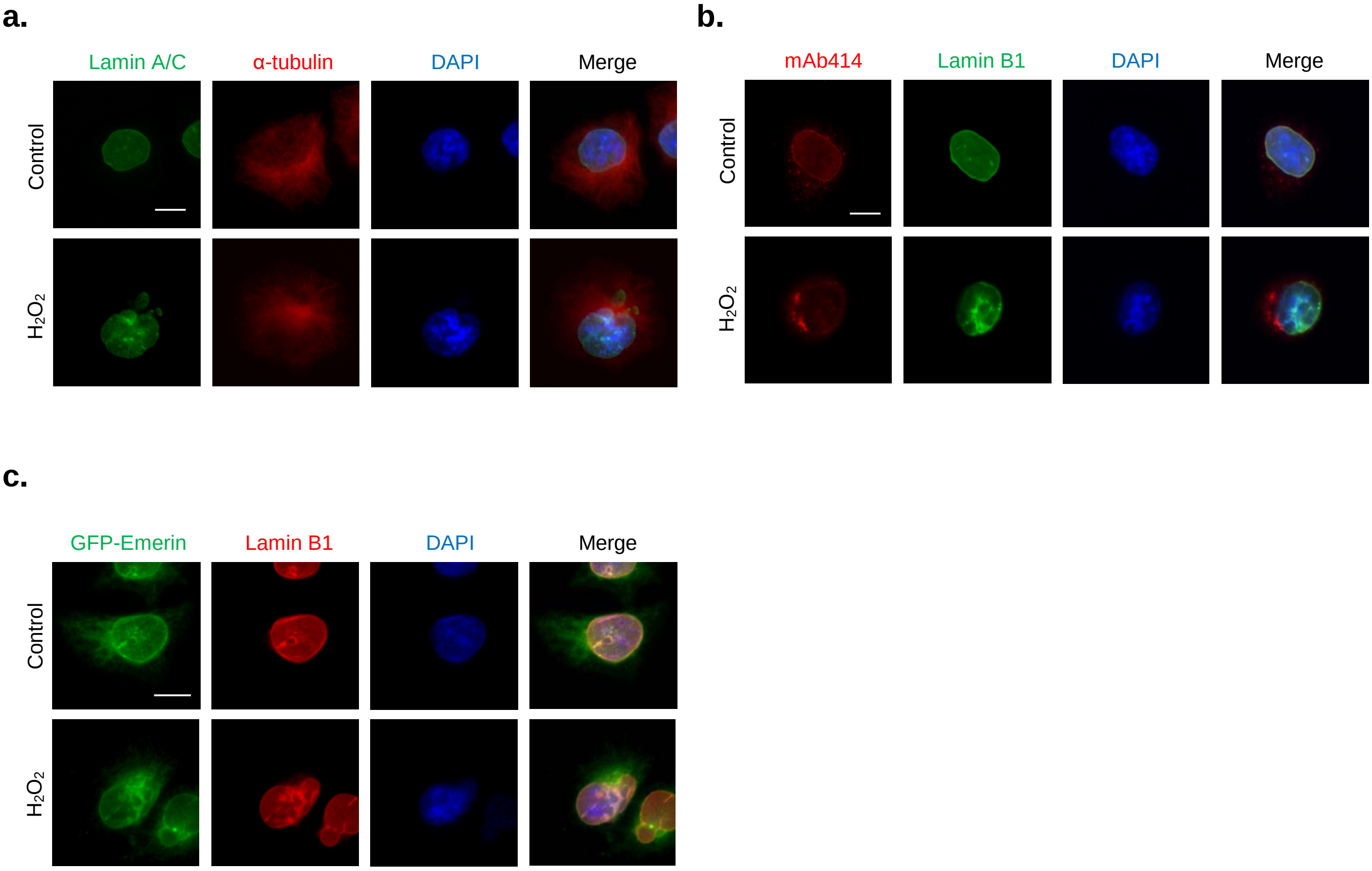

Supplementary Figure 3. Various elements constituting the nuclear membrane changes along with changes in nuclear shape. (a and b) Mitotic HeLa cells were treated with 100 μM H_2O_2 for 10 h, and structure of the nuclear envelope was assessed. Lamin A/C (a) and nuclear pore complexs (b) were visualized by using LaminA/C (green) and mAb414 (red) antibody, respectively. α-tubulin (red), Lamin B1 (green), DAPI (blue). Scale bar: 10 μm. (c) HeLa cells were transfected with expression vectors of GFP-Emerin, an inner nuclear membrane protein, and then mitotic cells were treated with 100 μM H_2O_2 for 10 h. Structure of nuclear envelope were visualized by using GFP (green) antibody. Lamin B1 (red), DAPI (blue). Scale bar: 10 μm.

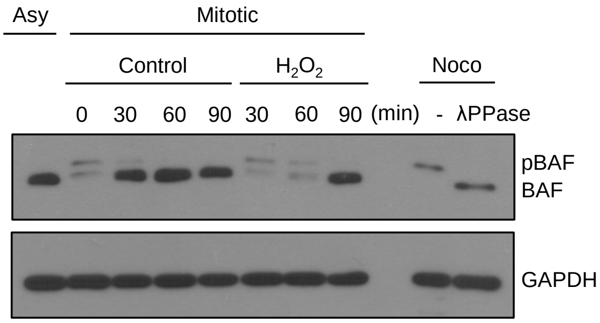

Supplementary Figure 4. H_2O_2 inhibits BAF dephosphorylation during mitosis. Mitotic cells were treated with 100 μM H_2O_2 and harvested in a time-dependent manner. To confirm the phosphorylation form of BAF, the mitotic HeLa cell extract obtained by nocodazole treatment was incubated with Lambda phosphatase. Proteins were analyzed by western blotting with the indicated antibodies.

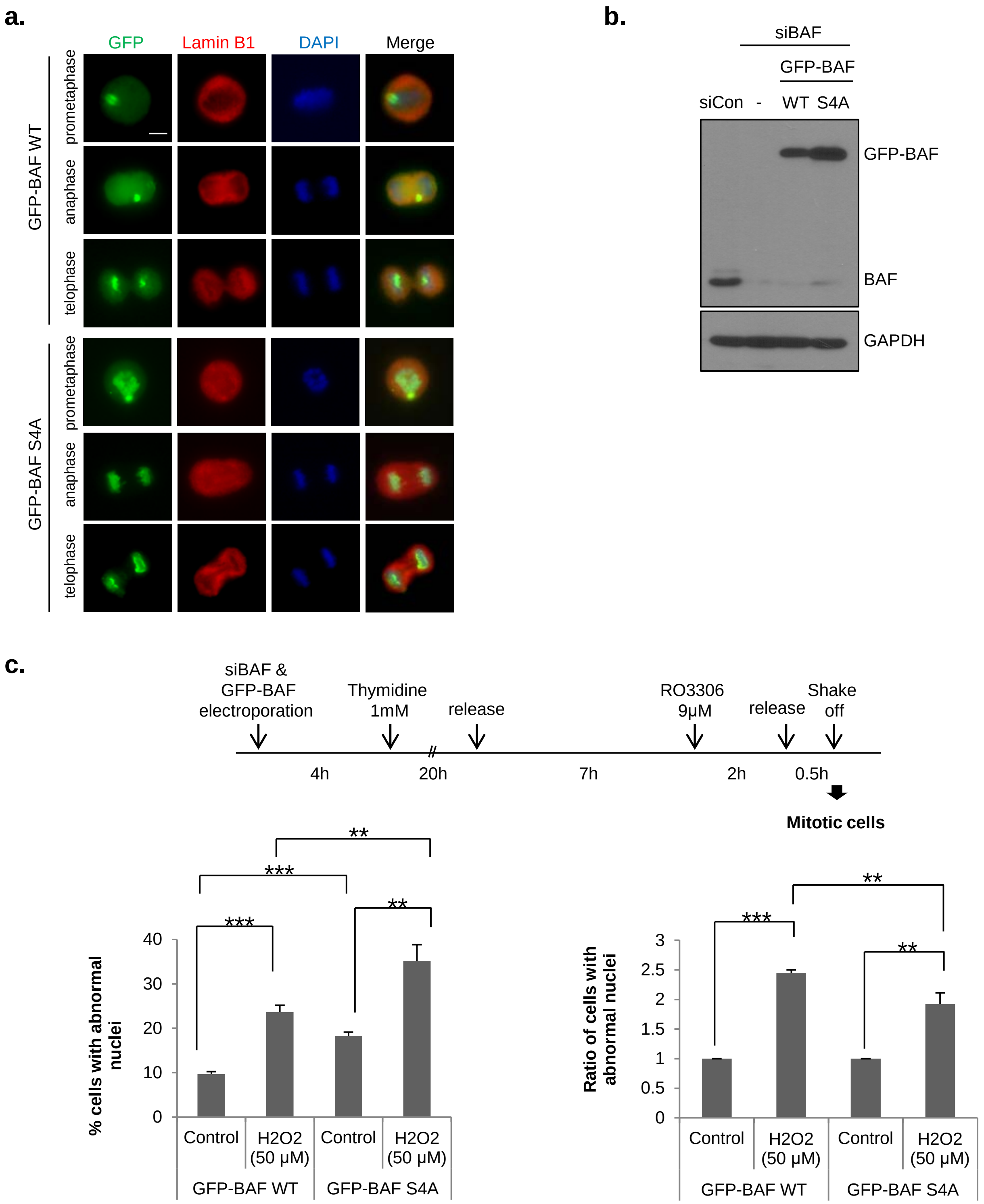

Supplementary Figure 5. BAF phospho-dead mutant partially restores H_2O_2-induced abnormal nucleation. (a) Left panel; HeLa cells were transfected with indicated vectors, and then mitotic cells were obtained by shake-off. BAF localization was visualized by using GFP (green) antibody. Lamin B1 (red), DAPI (blue). Scale bar: 10 μm. (b) Cells were transfected with indicated siRNAs and cDNAs simultaneously by using electroporation. After 36 h, asynchronous cell lysates were analyzed by western blot with the indicated antibodies. (c) Upper panel; experimental design to obtain mitotic cells. Lower panel; mitotic cells were treated with 50 μM H_2O_2 and nuclear shape was determined in cells expressing GFP-BAF after 10 h. The percentage of cells with abnormal nuclear shape was determined (left panel). Ratio of cells (H_2O_2-treated/non-treated control) with abnormal nuclear shape are shown (right panel). Results are shown as the ratio of mean ± SD from three independent experiments (n=100), **p<0.01, ***p<0.001 by Student's t-test.

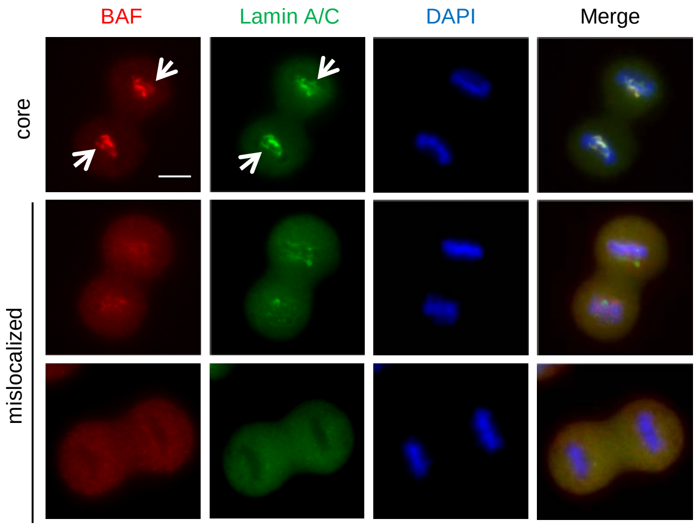

Supplementary Figure 6. Mislocalization of BAF is accompanied with mislocalization of lamin A/C. Mitotic HeLa cells were treated with 50 μM H_2O_2 for 30 min. And then, cells were immunostained for endogeneous BAF (red) and Lamin A/C (green). DAPI (blue). Arrow; BAF and Lamin A/C localized at the core region of telophase chromosome, Scale bar: 10 μm.
